# Introduction of Mismatches in a Random shRNA-Encoding Library Improves Potency for Phenotypic Selection

**DOI:** 10.1371/journal.pone.0087390

**Published:** 2014-02-03

**Authors:** Yongping Wang, Jacqueline S. Speier, Jessica Engram-Pearl, Robert B. Wilson

**Affiliations:** 1 Department of Pathology and Laboratory Medicine, Perelman School of Medicine at the University of Pennsylvania, Philadelphia, Pennsylvania, United States of America; 2 Department of Pathology and Laboratory Medicine, Children’s Hospital of Philadelphia, Philadelphia, Pennsylvania, United States of America; University Hospital Zurich, Switzerland

## Abstract

RNA interference (RNAi) is a mechanism for interfering with gene expression through the action of small, non-coding RNAs. We previously constructed a short-hairpin-loop RNA (shRNA) encoding library that is random at the nucleotide level [1]. In this library, the stems of the hairpin are completely complementary. To improve the potency of initial hits, and therefore signal-to-noise ratios in library screening, as well as to simplify hit-sequence retrieval by PCR, we constructed a second-generation library in which we introduced random mismatches between the two halves of the stem of each hairpin, on a random template background. In a screen for shRNAs that protect an interleukin-3 (IL3) dependent cell line from IL3 withdrawal, our second-generation library yielded hit sequences with significantly higher potencies than those from the first-generation library in the same screen. Our method of random mutagenesis was effective for a random template and is likely suitable, therefore, for any DNA template of interest. The improved potency of our second-generation library expands the range of possible unbiased screens for small-RNA therapeutics and biologic tools.

## Introduction

Small, non-coding RNAs can inhibit gene expression through interaction with mRNAs in a process called RNA interference (RNAi). In the canonical, post-transcriptional pathway, microRNAs (miRNAs) transcribed from the genome are processed by the ribonucleases Drosha and Dicer into ∼22-nucleotide (nt) small-interfering RNAs (siRNAs). The RNA-Induced Silencing Complex, RISC, uses the siRNAs to cleave and/or inhibit the translation of complementary mRNAs in a sequence-specific manner [2]. Increasing evidence also points to roles for these non-coding RNAs in nuclear RNAi, transposon regulation, chromatin epigenetics, and overall genomic stability [3]. Most endogenous miRNAs that have been described target short sequences in the 3′ untranslated regions (UTRs) of not a single mRNA, but a large number of mRNAs simultaneously [4], anchored by a “seed” region of approximately six nucleotides (miRNA guide-strand nucleotides 2–7) supplemented with either a U at position 1 or a target match at position 8 [5]. Many miRNAs that target coding regions, including exon-exon junctions, have also been described; taken together, these findings suggest that mutations in miRNA target sites heretofore considered “silent” might have phenotypic consequences [6]. Underscoring the complex nature of miRNAs, some have been reported to *activate* gene expression by targeting promoter regions of certain genes [7], [8]. In addition, three independent miRNAs targeted to the 3′ UTRs of three different mRNAs repressed translation in proliferating cells but activated translation in cell-cycle-arrested cells [9].

RNAi libraries based on canonical RNAi have been developed for screening purposes. Most of these libraries were designed to encode shRNAs that target single, specified genes with multiple constructs to ensure adequate silencing [10], [11], [12], [13], [14]. In part to decrease costs associated with generating thousands of individual constructs by computer-aided design, some investigators have used enzyme-based approaches to construct RNAi libraries from either cDNA or genomic DNA fragments [15], [16], [17], [18], conferring a certain degree of randomness to sequences in the library. These RNAi libraries are designed to identify single genes of biologic interest, or genes that encode potential targets for conventional drug development. However, for identifying shRNAs or siRNAs to be used in and of themselves as therapeutics or biologic tools, the most effective sequences may target many genetic elements and/or may act through non-canonical mechanisms. To identify such sequences, libraries that are random at the nucleotide level, and therefore unbiased with respect to mechanism of action, are preferable.

We previously described the synthesis of a completely random shRNA-encoding library with 29-mer complementary random sequences at the stem, linked by a non-complementary loop. We demonstrated proof of principle by isolating hit sequences that protect an IL3-dependent cell line, FL5.12, from IL3 withdrawal, and we successfully optimized one of our hit sequences by random mutagenesis (to create a sub-library) and re-screening [1]. Other groups have also produced random RNAi libraries: one uses two opposing promoters to transcribe linear RNAs from the same 19-base-pair random sequence simultaneously, resulting in an siRNA-encoding library [19]. This approach has several limitations: 1) siRNAs are less potent and have shorter life spans in cells than shRNAs [20], [21]; in the context of screening random libraries, potency is critical since the initial effects are expected to be weak. 2) Its design precludes mismatches in the RNA duplex, a factor contributing to the potency of endogenous miRNAs. 3) shRNAs or siRNAs with 27–29 bp stems are more potent inducers of RNAi than constructs with 19–21 bp stems [22], [23]; however, siRNAs >23 bp in length are more likely to induce non-specific interferon responses [24]. Another group used a recombinase technology to generate an shRNA-library similar to ours, but the design also precluded the introduction of mismatches in the RNA duplex [25].

In pooled, phenotypic screens of random shRNA libraries, the potency of initial hit sequences is critical: The phenotypes of cells in pooled cultures exhibit a natural variation depending on confluence, cell cycle, edge effects, etc., and the phenotypic effects of initial hit sequences may be weak relative to that variation. Another concern is retrieval of hairpin-loop structures by PCR, which can be difficult to amplify. To overcome both of these problems, we attempted to construct a second-generation library that incorporates mismatches between the two halves of the shRNA stem, a non-trivial task given the constraints of our library synthesis. Herein we describe an approach, based on the work of Lehtovaara *et. al.* [26], to incorporate random mutations in the two halves of the complementary stem-encoding sequences of our random library. We tested whether our second-generation library was indeed more potent than our first-generation library by comparing their performances in the same screen.

## Results

### Library Synthesis

Our library design precludes methods of random mutagenesis based on PCR. Non-PCR methods that have been described include chemical mutagenesis of bases, with ethyl methane sulfonate (EMS) [27], [28], nitrous acid, formic acid, or hydrazine [29]. Other methods use so-called “universal bases,” such as inosine or novel synthetic bases, capable of pairing with any of the natural bases [30], [31]. However, the pairing preferences of these bases for the natural bases have never been optimal [32], [33], [34]. Furthermore, all of the described methods are based on mutagenesis of a known, fixed sequence, whereas our target is completely random. Based on these aforementioned methods, multiple attempts to mutagenize random target sequences, in the context of our library synthesis procedure, were unsuccessful (data not shown).

**Figure 1:**
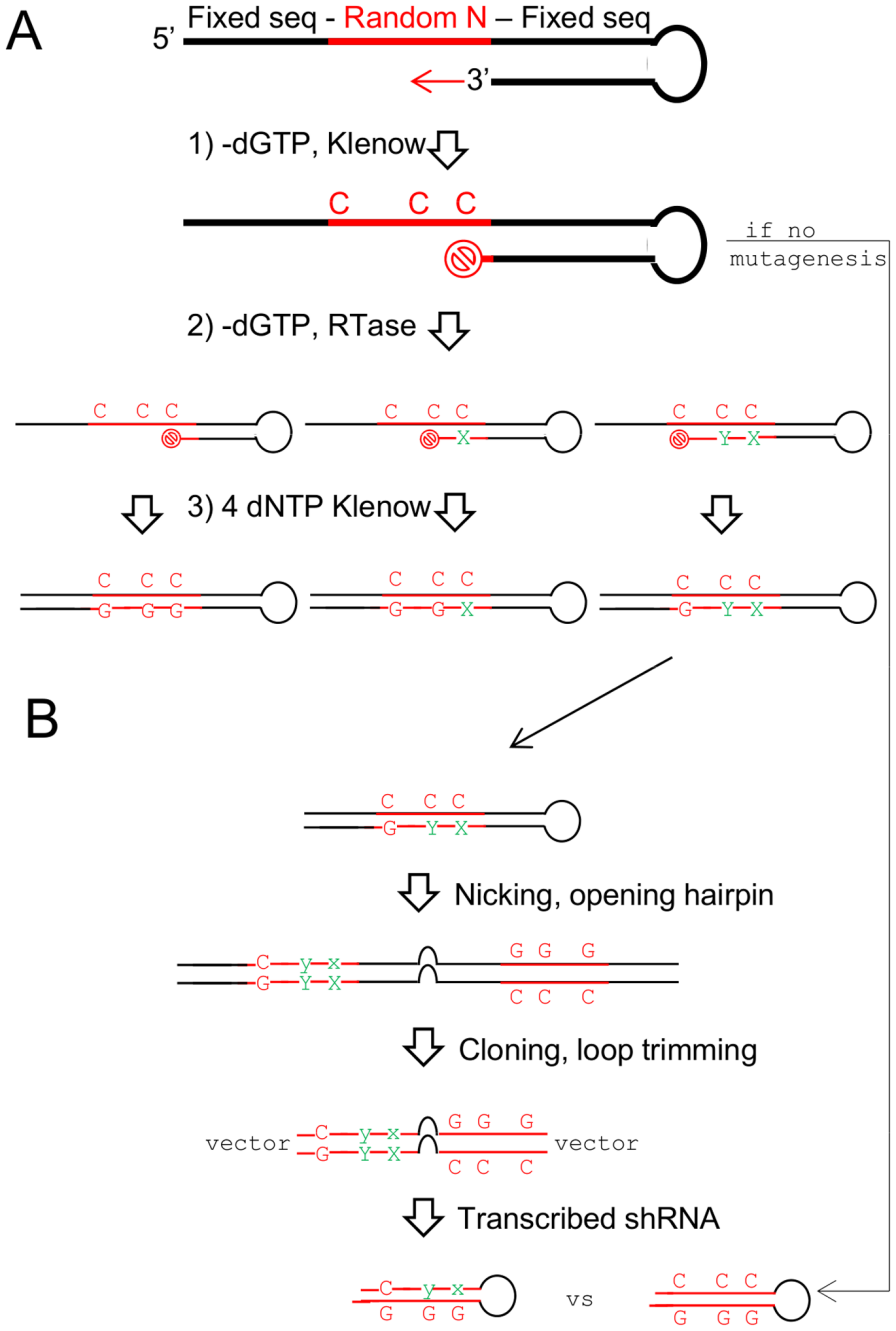
Introduction of mismatches by random mutagenesis. (A) Three steps were used to introduce mutations into the random template. **Step 1**: Extension reaction, minus one of the four dNTPs; in this example, minus dGTP. The extension should in theory stop at the first C. **Step 2**: Error prone reverse transcription forcefully incorporate a mismatched base opposite the C, still minus dGTP, but with different ratios of dATP, dCTP, and dTTP to compensate for their different paring affinities with C. Depending on the length of incubation, different lengths of stalled fragments will result. **Step 3**: After mutations are introduced, the extension reaction is completed with all four dNTPs present. (B) Abbreviated depiction of the rest of the library synthesis (described in detail previously [1]). Briefly the (single-stranded) DNA is nicked near the 5′ end, the hairpin is opened with an extension reaction using a strand-displacing polymerase, the ends are digested for cloning, the loop is digested asymmetrically and re-ligated to form a final loop sequence of 6 nucleotides (5′-CTAAAC’-3). For comparison, a non-mutagenized hairpin is also shown.

Our eventual approach was based on the work of Lehtovaara *et. al.* [26] (Materials and Methods, Figure 1A). Briefly, the first step involves four independent DNA-polymerase extension reactions, with each extension lacking one of the four deoxyribonucleotide triphosphates (dNTPs). Figure 1A depicts the extension reaction lacking dGTP; theoretically, the polymerase should stall at the first template base whose complementary dNTP is missing, in this case at “C”. The second step uses an error-prone polymerase to forcefully incorporate the wrong base where the initial polymerase stalled; the concentrations of the other three dNTPs are included at ratios that compensate for their differential pairing affinity with the template base. (The depiction of the second step in Figure 1A is only schematic since it shows the same template generating three differently stalled extension reactions; however, the number of possible random 29-mer templates –4^29^– mathematically precludes the chance of the same template being present twice at the reaction scale we used, hence in actuality each template can be mutagenized only once.) The third step is a final extension reaction with all four dNTPs.

The rest of the library synthesis was essentially as described previously [1] and is shown schematically in Fig. 1B. Each clone comprises a 29-nucleotide random sequence and its reverse complement in the same strand of DNA, separated by a non-complementary loop sequence (5′-CTAAAC-3′). In addition to the introduction of random mismatches between the two halves of the stem-encoding sequences, we increased the complexity of our second-generation library by 10-fold, from 300,000 clones in our first-generation library [1] to 3 million clones. We also changed the fluorescent reporter from Green Fluorescent Protein (GFP) to the Red Fluorescent Protein mCherry, which allows our library to be used with GFP reporter constructs in gene-activation screens.

### Library Characterization

**Figure 2:**
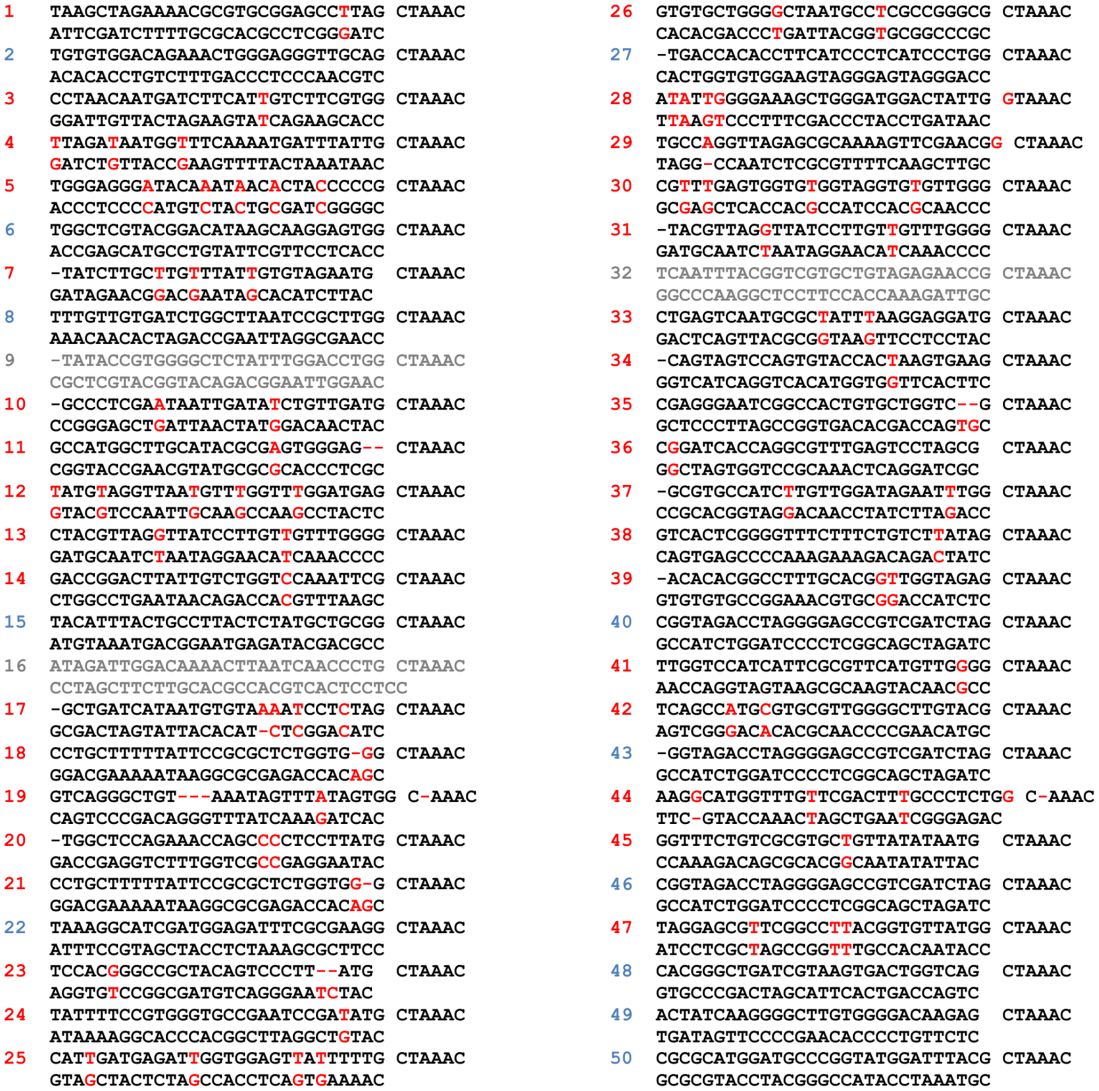
Sampling of 50 sequences from the second-generation library. Out of 50 sequences sampled randomly, 35 (numbered in red, 70%) have mismatches, 12 (numbered in blue, 24%) have no mismatches, and 3 (numbered in gray, 6%) have non-complementary stem sequences and would not be expected to form a hairpin structure.

We sequenced 50 random clones from our second-generation library (Fig. 2). Thirty-five (70%) have mismatches between the two halves of the stem, 12 (24%) lack mismatches, and three (6%) have (essentially) non-complementary halves and would not be expected to encode shRNAs. (Of the many clones sequenced from our first-generation library, we never observed clones with non-complementary halves.) Among the different types of mismatches, T-G is the most common, even with the intentional skewing of the three dNTPs in step 2 (Table 1).

**Table 1:** List of mismatches from the 50 clones sequenced from the three-million-clone, second-generation library.

Mismatch (descending order)	Total	%
TG	30	45
TT	10	15
AC	5	7.5
GT	5	7.5
AG	4	6
GG	4	6
CC	3	4.5
TC	2	3
AA	1	1.5
GA	1	1.5
CA	1	1.5
CT	0	0

We observed unexpected deletions (clones 11, 17–19, 21, e.g.), as well as deletions/mutations occurring in the loop sequence (clone 19, 28, 44). Also, some clones “skipped” mutations where we would have expected them to occur. Based on Figure 1, the method should introduce mutations starting with the first available G, and potentially every following G (depending on the length of incubation with the reverse transcriptase). However, this rule was not always followed (Fig. 2). For example, in clone 4, the first template G in the extension reaction was matched with a C, whereas the following Gs were mismatched with Ts, as expected. In clone 13, a T-A match is flanked by two mismatches (T-G and T-T).

Our library was designed to encode 29-bp stems. Both our first- and second-generation libraries contain occasional clones with 28- and 30-bp stems (Fig. 2 and data not shown), probably due to 1-nt errors in the length of the original template oligo. Both libraries also contain occasional clones with 28-nt-29-nt stems, probably due to the inherent imprecision of the downstream-cutting enzyme *BtgZ* I in one of the initial steps of the library synthesis [1]. Assuming that most of the mismatches and deletions arose from the mutagenesis process in the construction of our second-generation library (and not from the original oligo template), we observed ∼80 mistakes in ∼1400 positions from 50 clones sequenced, leading to an estimated mutation rate of ∼5.7%.

### Library Validation

As with our first-generation library, we validated our second-generation library by packaging the library as retroviruses and screening for shRNAs that protect the IL3-dependent, murine pro-B cell line FL5.12 from IL3 withdrawal. After 2–3 days in the absence of IL3, ∼100% of FL5.12 cells die by apoptosis; if Bcl-xL is expressed, >90% of the cells are rescued [35]. To minimize the chance that a weak hit sequence would be diluted by inactive shRNAs, we aimed to achieve 30% infectivity, thereby ensuring that most cells would express only one shRNA. Consistently lower infectivity with mCherry vectors than with GFP vectors suggested that mCherry is slightly more toxic to FL5.12 cells. In the end, we infected ∼150 million FL5.12 cells to ∼6% mCherry positivity (∼9 million infected cells), ensuring adequate coverage of our three-million-clone, second-generation library. Cells infected to ∼10% mCherry positivity with a single, randomly selected shRNA were used as a control.

To compare the first- and second-generation libraries directly, we screened both libraries, side by side. We enriched for true positives by subjecting the cells to repeated cycles of withdrawal from IL3, followed by recovery in media with IL3 (Materials and Methods). Whereas previous hit sequences from the first-generation library were isolated by withdrawing IL3 for three days per cycle [1], we performed the side-by-side comparison screens using both three-day and four-day withdrawals from IL3, having hypothesized that the mismatches introduced into the second-generation library would increase biological activity. As expected with the presence of hit shRNAs, the percentage of fluorophore-positive cells started to increase after 3–4 cycles in all four arms of the experiment (Fig. S1). In both the three-day- and four-day-cycle experiments with the second-generation library, the mCherry percentage stopped increasing in later cycles (Fig. S1), most likely due to the emergence of mCherry-negative, IL3-independent clones.

**Figure 3:**
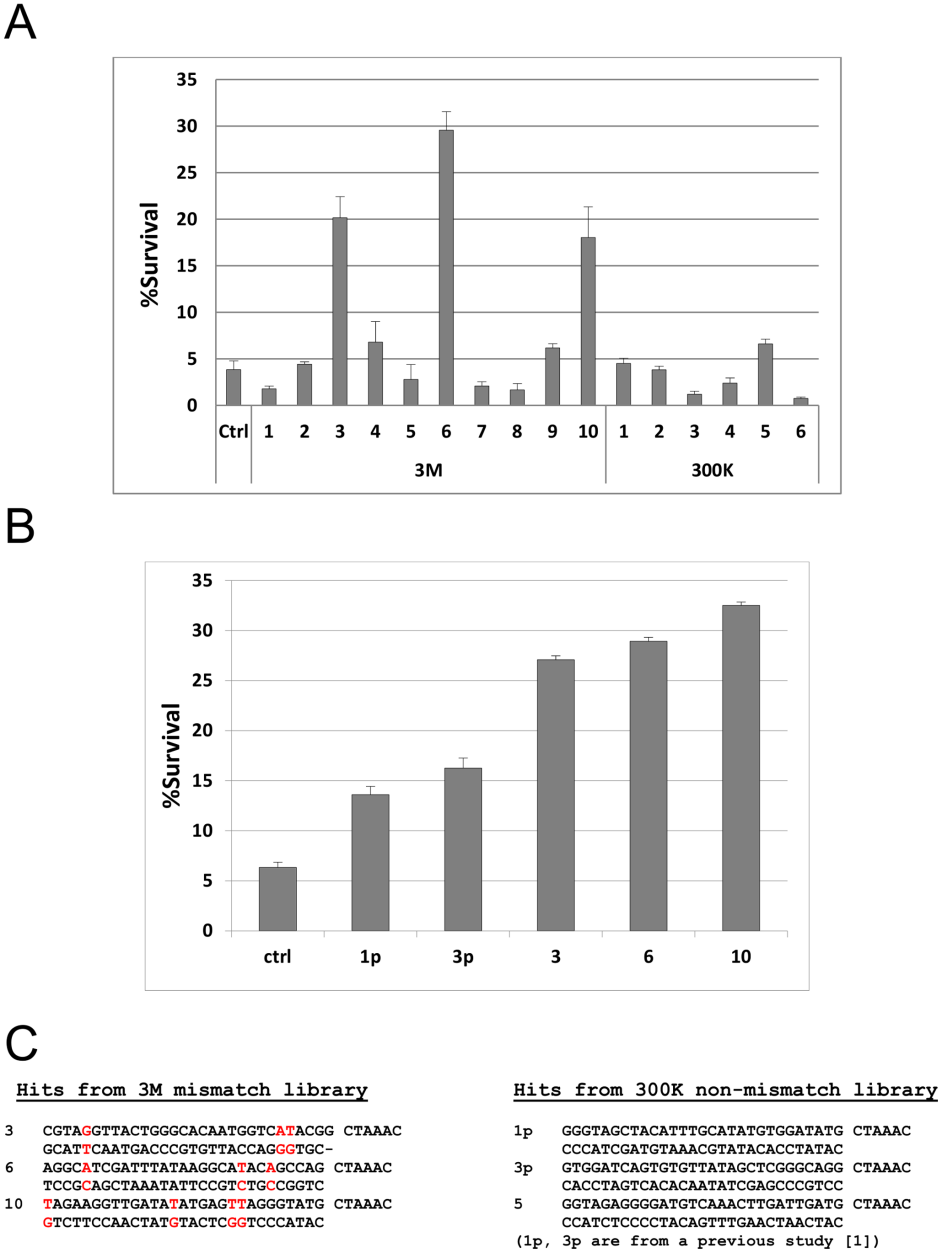
shRNAs selected from the second-generation library better protect FL5.12 cells from IL3 withdrawal. (A) FL5.12 cells were transduced with different shRNA clones isolated from the side-by-side screens of the first-generation (300K) and second-generation (3M) libraries. The cells were subjected to an overnight IL3 withdrawal. Survival percentages (percentages of GFP+/To-Pro-3- cells) are shown, relative to the beginning of IL3 starvation. The six clones offering the most protection, relative to a control shRNA, were clones 3M-3 (p<0.0001), 3M-4 (p = 0.10), 3M-6 (p<0.0001), 3M-9 (p = 0.019), 3M-10 (p<0.0001) and 300K-5 (p = 0.011). Three clones from the second-generation library (3M-3, -6, and -10) were all significantly more protective than clone 300K-5 (p<0.0001 for all three). (B) Clones 3M-3, -6, and -10 were compared to two hit shRNAs (1p and 3p) isolated in our previous study from the first-generation library. The improved survival was highly statistically significant, with p<0.0001 by Student’s t-test in pair-wise comparisons between any of the three clones (3M-3, -6, or -10) versus either 1p or 3p. (C) Sequences of clones 3M-3, -6, and -10 from the second-generation, mismatched library, and of clones 1p, 3p and 300K-5 from the first-generation, non-mismatched library.

We harvested cells at their respective peak percentages of fluorophore-positive cells, isolated genomic DNA, amplified the shRNA-encoding cassettes by PCR, and cloned back into pSiren/GFP. Randomly selected clones enriched from both the first- and second-generation libraries were tested side-by-side against a control random shRNA (Fig. 3A). Of the six clones tested from the first-generation library, only one was active (and only slightly) in protecting the cells from IL3 withdrawal, whereas of the 10 clones tested from the second-generation library, two were slightly active and three were highly active (Fig. 3A). The active clones from both libraries were tested again and similar results were obtained (Fig. S2). We also tested the three highly active hit clones from the second-generation library against the most active hit clones isolated from the first-generation library in our previous study [1] (clones “1p” and “3p”), and all three of the clones from the second-generation library were significantly more active (Fig. 3B), though their relative activities varied somewhat from experiment to experiment (Fig. 3A, Fig. 3B, and data not shown). However, these three clones consistently offered an approximately four-to-five-fold survival advantage relative to a random control clone, whereas hit clones from the first-generation library, both from our earlier study and from the side-by-side comparison performed herein, offered an approximately two-fold survival advantage. The sequences of the three highly active clones are shown in Figure 3C, alongside the sequences of 1p, 3p and the slightly active clone from the direct-comparison screen with the first-generation library.

To date, of the more than 20 clones we have retrieved after biologic selection and PCR retrieval, *all* of them had mismatches between the two halves of the stem, whereas the mismatch percentage in 50 sequenced clones from the library itself was ∼70% (Fig. 2), again consistent with our hypothesis that the introduction of mismatches increased potency and/or retrieval efficiency. Among all the hit sequences identified from the two libraries, strong or weak, there were no obvious sequence similarities.

## Discussion

We have constructed a three-million-clone, shRNA-encoding library that is completely random at the nucleotide level, with mismatches between the two halves of the stem-encoding sequences. Our library allows for unbiased phenotypic selection of shRNA sequences to be used as shRNA or siRNA therapeutics or biologic tools. We previously constructed a 300,000-clone library, without mismatches, and identified shRNAs that double the survival of FL5.12 cells upon IL3 withdrawal. We expected the initial hit sequences to be weak since shRNAs are processed to ∼22-nucleotide guide strands and there are ∼18 trillion possible 22-mer sequences (4^22^), whereas our first-generation library comprises only ∼300,000 clones. However, the number of possible seed sequences, which are sufficient for partial RNAi, is on the order of 16,000, and thus even our first-generation library is likely to include virtually all of them.

In our previous study, we optimized one of our first-generation hit sequences by random mutagenesis and re-screening [1], random mutagenesis for hit-optimization being much more straightforward than the method of random mutagenesis to create mismatches between the two halves of the shRNA stem, described herein. An analysis of the optimized sequence showed that the potency was improved in part by the introduction of a mismatch between the two halves of the stem [1], which is consistent with the finding that mismatches between the two halves of the stem facilitate the loading and unwinding of the RNA duplex in RISC [36], [37]. In fact, all endogenous miRNAs we have examined in the Sanger database (www.mirbase.org) carry such mismatches. Thus, we hypothesized that introducing mismatches into our random library would increase the potency of initial hits. Another concern regarding our approach is retrieval of hit sequences, which tend to amplify poorly because of their stem-loop structures. We hypothesized that the introduction of mismatches between the two halves of the stem-encoding sequences in our second-generation library would improve the the efficiency of retrieval by PCR, thereby expanding the range of feasible phenotypic screens.

Consistent with our first hypothesis, the introduction of mismatches between the two halves of the stem-encoding sequences significantly improved the potency of initial hit sequences when compared with our first-generation library in the same screen: Whereas initial hit sequences from the first-generation library doubled survival of FL5.12 cells after IL3 withdrawal, initial hit sequences from the second-generation library quadrupled and quintupled survival relative to a random control clone. Consistent with our second hypothesis, retrieval of hit sequences was apparently more efficient: Whereas ∼70% of the second-generation library sequences have mismatches, *all* of the sequences we retrieved at the end of the screen had mismatches. In addition, the sequencing of clones without mismatches (in our sequencing core facility) often stalls part way through the 29-nucleotide stem (presumably due to the hairpin-loop structure); reading through the entire 29-nucleotide stem was successful at a noticeably higher frequency in sequencing clones with mismatches.

Our random-mutagenesis methodology for creating mismatches is not perfectly random since each specific template molecule will be mutated at only one of four bases, depending on whether that specific template molecule ends up in the tube lacking A, C, T, or G. In addition, even if two identical template molecules ended up in the same tube, the first instance of a base whose complementary dNTP is missing is likely mutated at a different frequency than that of the second instance of the same base. Fortunately for us, the infidelity of the M-MuLV reverse transcriptase was greater than we expected, and more types of mutations (including deletions) were introduced than were predicted theoretically. T-G mismatches were most common (despite the fact we used the least amount of T in the reaction lacking C), likely due to the fact that G and U can form a wobble base pair in RNA and T possesses the same G-pairing –NH and  = O groups as U possesses. Further refinements could be made by adjusting the ratios of different dNTPs; however, in making our shRNAs more biologically active, and technically easier to manipulate, the mutations we introduced serve our purposes.

The primary advantages of our random shRNA approach are that it is unbiased with respect to mechanism(s) of action, of which our understanding remains incomplete, and that it leverages the capacity of small RNAs to alter the expression of many genes simultaneously. A concomitant disadvantage, which we have discussed previously [1], is that elucidation of the precise mechanisms of action of hit shRNAs is difficult. We have used both gene-expression profiling and existing miRNA target-identification algorithms to identify putative targets. But it is unclear whether, or how much, each putative target contributes to the phenotype. Cases in which putative targets cluster significantly in certain pathways may allow us to narrow down to a primary contributor to the screening phenotype. However, because of the random design of our library, and because seed sequences are sufficient for partial effects, we believe that in most cases no single gene or pathway will explain the screening phenotype. It is also plausible that some of the activities of these shRNAs are through mechanisms other than canonical RNAi; for such activities, the benefit of having mismatches between the two halves of the stem is uncertain.

Among the hit shRNAs we have identified, none show any discernable sequence homologies, either overall or in the seed sequences (assuming canonical RNAi). We have experimental evidence that clones 1p and 3p protect FL5.12 cells from IL3 withdrawal through very different mechanisms (manuscript in preparation), but that evidence is indirect and not based on a detailed knowledge of canonical targets. Our library complements existing single-gene-targeting RNAi libraries, and serves a different purpose. We seek to identify small RNAs to be used as therapeutics or biologic tools in and of themselves, with or without fully identifiable mechanisms of action. Our approach is functional in that we allow the cells to tell us which sequences are most effective, and least toxic, without prior assumptions. Although we are attempting to further elucidate mechanisms of action for our hit shRNAs, our primary goal is to achieve useful therapeutic and biologic phenotypes with minimal toxicity.

Delivery of shRNA and siRNA therapeutics to specific tissues remains a challenge, but is a very active area of research and is increasingly being solved with such approaches as lipid nanoparticles, peptide-conjugates, aptamers, and other innovations [38], [39], [40]. Potential applications are numerous, including protection against infectious agents, reversal of cellular defects associated with genetic disorders, and the control of cellular differentiation states. Any cellular system with a selectable phenotype, such as survival, enhanced growth, or a flow-sortable marker, and with a reasonable signal-to-noise ratio, is amenable to our approach. We are currently screening our library in some of these systems. We are also pursuing what we call negative selection, in which we seek to identify shRNA sequences that are lost from a pool. The primary goal of negative selection in our laboratory is to identify shRNAs that are *selectively* toxic to cells with cancer-associated mutations, thereby improving therapeutic indices. Our improved, second-generation, random shRNA-encoding library increases the likelihood of success in identifying biologically and therapeutically useful small RNAs.

## Materials and Methods

### Random Mutagenesis

A 132-mer oligo, which can form an internal partial hairpin, was synthesized by ChemGenes (Fig. 1A): 5′CCCTATATGCATGCTGAGGAAGAATTCAGCGGCCGCGATGACCTGAAA*A*A*N*N*NNNNNNNNNNNNNNNNNNNNNNNNNNGGTTTAAACAGGTGAGAATTCTATTCAGTCATAGAATTCTCACCTGCTTAAAGC-3′. The asterisks represent thio-ester bonds. The details of the three mutagenesis steps illustrated in Figure 1A are shown below. The individual dNTPs are from Denville Scientific, and all buffers and enzymes are from New England Biolabs. Numbers listed in the steps below represent microliters unless indicated otherwise. Minus signs after nucleotides indicate that they are dropped out of the indicated extension mix.

**Table:** 

**Step 1**:	A- C- G- T-			
132-mer (0.1 nmole/µl)	1	1	1	1
Water	14.5	14.5	14.5	14.5
dATP (100 mM)	–	0.5	0.5	0.5
dCTP (100 mM)	0.5	–	0.5	0.5
dGTP (100 mM)	0.5	0.5	–	0.5
dTTP (100 mM)	0.5	0.5	0.5	–
Boil 3 min, quick spin, cool to 37°C				
NEB Buffer 2	2	2	2	2
Klenow (exo-) (5U/µl)	1	1	1	1
37°C x 30 min, followed by ethanol precipitation of DNA.				
**Step 2**:	A- C- G- T-
Mix components below first to a total volume of 95 µl, and use the mix to resuspend the DNA pellet from step 1.				
Water	82	82	82	82
NEB RTase buffer	10	10	10	10
Adjust each stock concentration such that when 1 µl is used, final concentrations are:				
dATP	–	1 mM	0.1 mM	0.5 mM
dCTP	1 mM	–	1 mM	0.5 mM
dGTP	.01 mM	0.2 mM	–	1 mM
dTTP	1 mM	0.002 mM	0.2 mM	–
After resuspending pellet, add M-MuLV reverse transcriptase (RTase, NEB, 200U/µl)				
RTase volume	2.5	2.5+2.5	2.5+2.5	2.5+2.5
RTase incubation at 42C	1 hr	2+2 hr	1+1 hr	1+1 hr
2.5+2.5/2+2 hr means that 2.5 µl of RTase is incubated for 2 hrs at 42°C and another fresh 2.5 µl is added for another 2 hrs at 42°C. DNA is again ethanol precipitated.				
**Step 3**:	A- C- G- T-			
Mix components below first for a total volume of 50 µl, and use that mix to resuspend the DNA pellet from step 2.				
Water	41	41	41	41
dNTP (10 mM each)	2	2	2	2
NEB Buffer 2	5	5	5	5
Klenow (exo-) (5U/µl)	2	2	2	2
37°C for 30 min.				

The remainder of the library synthesis is carried out as described in the making of the non-mismatch library [1]. As in that library, the current oligo has a G at the end of the N29 random segment (i.e., the segment is N28+G), representing 1/4 of the complete, random N29 library that can be made similarly using N28+A, N28+C, and N28+T.

### Cell Culture, Retroviral Transduction

The FL5.12 pro-B cell line [41] was a gift from Dr. Craig Thompson (Memorial Sloan-Kettering Cancer Center). FL5.12 cells were cultured in RPMI 1640 media with 10% FBS (Thermo Scientific), 10 mM Hepes pH 7.4, 100 U/ml Penicillin, 100 mg/ml Streptomycin, 55 mM β-Mercaptoethanol (all from Gibco), supplemented with 0.6 ng/ml IL3 (BD Pharmingen). To prepare retroviral supernatant for infection, 293T cells at ∼70% confluency were transfected with Effectene reagent (Qiagen) according to manufacturer’s instructions. The pSiren (Clontech) library was co-transfected with an ecotropic retroviral packaging plasmid pCL-Eco (Imgenex) at a dose of 2.5 µg total DNA per well in a 6-well plate. Supernatant was harvested to infect FL5.12 cells with 3 cycles of centrifugation (2500 g for 45 minutes) and incubation (2 hrs), in the presence of 5 µg/ml polybrene (Sigma). Infection efficiency was monitored by mCherry expression on a BD LSRII flow cytometer. Ideally the mCherry percent positivity was kept at ∼33% or less whenever a library was used to transduce cells, so that, by Poisson distribution, the majority of the infected cells received only one construct.

### Sequence Enrichment

To enrich for sequences that support cell survival during IL3 withdrawal, infected cells were subjected to cycles of IL3 withdrawal and recovery. In each cycle, apoptosis was induced in FL5.12 cells by washing three times with IL3-negative medium and resuspending in IL3-negative medium. After 72 or 96 hours cells were resuspended in medium containing IL3 to recover. No attempts were made to get rid of dead cells during this process. The cycling was repeated until the mCherry or GFP percentage of the FL5.12-cell population enriched to at least 2-fold higher than the post-infection percentage.

### Sequence Retrieval

To retrieve shRNA-encoding sequences, cells that have been enriched for mCherry after IL3 starvation/recovery cycles were pelleted, and their genomic DNA was extracted using QIAamp® DNA Mini Kit (Qiagen). The shRNA-encoding cassette was amplified from genomic DNA using the following protocol: 95°C for 5 min, 95°C/56°C/72°C at 30 s/45 s/2 min for 30 cycles, and 72°C x 10 min, using Vent® exo- DNA polymerase (NEB) and 6 mM MgSO_4_ with primers flanking the shRNA-encoding cassette on the vector pSiren. The sequences of the primers are 5′-CCGGAATTGAAGATCTGGG-3′ and 5′-CCGTAATTGATTACTATTAATAACTAGAATTC-3′. Products amplified by Vent were subject to another round of amplification using fresh dNTPs and Bst DNA polymerase (NEB) by using the following protocol: before adding Bst, 95°C for 5 min, 65°C for 30 s; add Bst, 65°C for 30 min. Retrieved sequences were digested with *Bgl* II and *EcoR* I, and ligated into pSiren (GFP).

### Hit Confirmation

Individual clones retrieved as described above were tested in FL5.12 cells for their ability to protect against IL3 withdrawal, against control, and against previous hit sequences (all in pSiren/GFP). Apoptosis was induced by washing three times with IL3-negative medium and resuspending in IL3-negative medium. Tests of individual clones were carried out with an overnight IL3 starvation of 22–26 hours. Cells were then stained with 10 nM To-Pro-3 iodide (Invitrogen). The percentage of GFP-positive (infected) and To-Pro-3-negative (live) cells relative to the start of the experiment (just prior to the IL3 withdrawal) were determined by flow cytometry on a BD FACSCalibur. Confirmed hits were then sequenced using the PCR primers.

### Statistical Analysis

Pair-wise comparisons of means were conducted using Student’s t-test. Error bars represent standard deviations. The data points for each bar graph were determined from 3 to 4 independent experiments.

## Supporting Information

Figure S1 **GFP and mCherry percentage after IL3 starvation/recovery cycles.** FL5.12 cells were screened side-by-side with transduction of the first-generation (300K GFP) or second-generation (3M mCherry) library, along with the corresponding control shRNA. Cells were subject to IL3-withdrawal of three days (A) or four days (B). GFP or mCherry percentages after each recovery (Rec) are shown.
(PDF)

Figure S2 **shRNAs selected from the second-generation library better protect FL5.12 cells from IL3 withdrawal.** FL5.12 cells were transduced with different shRNA clones obtained from the side-by-side screen. The cells were subject to an overnight IL3 withdrawal. Survival percentages (percentages of GFP+/To-Pro-3- cells) are shown, relative to the beginning of IL3 starvation. All six clones, five from the second-generation (3M) library and one from the first-generation (300K) library were significantly more protective than control (p<0.0001 for all). Clones 3M-3, -6, and -10 were also significantly more protective than clone 300K-5 (p<0.001 for all).
(PDF)
