# Supplementary material for: Introduction of Mismatches in a Random shRNA-Encoding Library Improves Potency for Phenotypic Selection
Source: PLoS One. 2014 Feb 3;9(2):e87390. doi: 10.1371/journal.pone.0087390 (PMC3911983; doi:10.1371/journal.pone.0087390)
Supplement: Figure S1 — GFP and mCherry percentage after IL3 starvation/recovery cycles. FL5.12 cells were screened side-by-side with transduction of the first-generation (300K GFP) or second-generation (3M mCherry) library, along with the corresponding control shRNA. Cells were subject to IL3-withdrawal of three days (A) or four days (B). GFP or mCherry percentages after each recovery (Rec) are shown. (PDF) [file pone.0087390.s001.pdf]

A

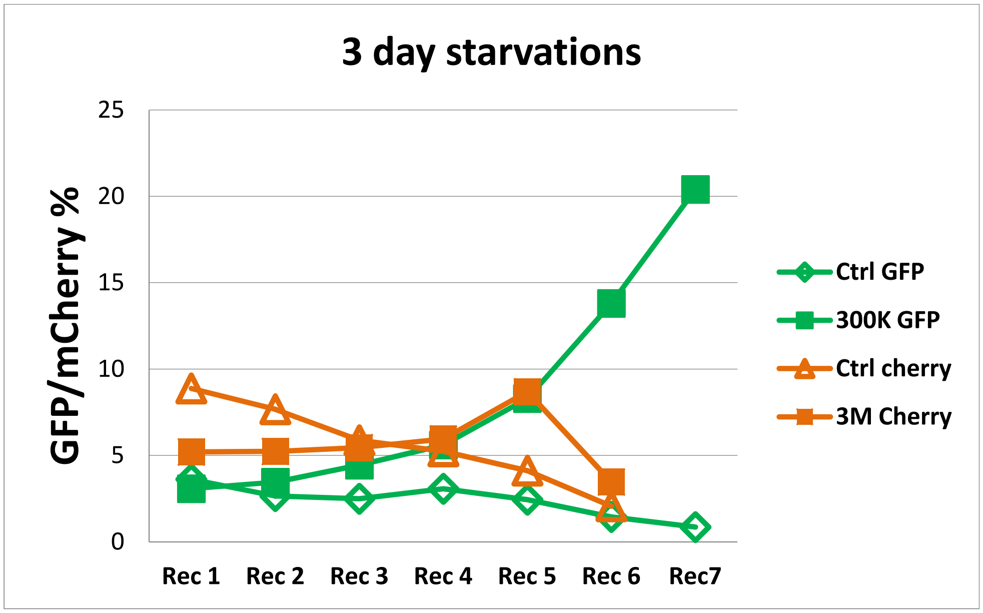

B

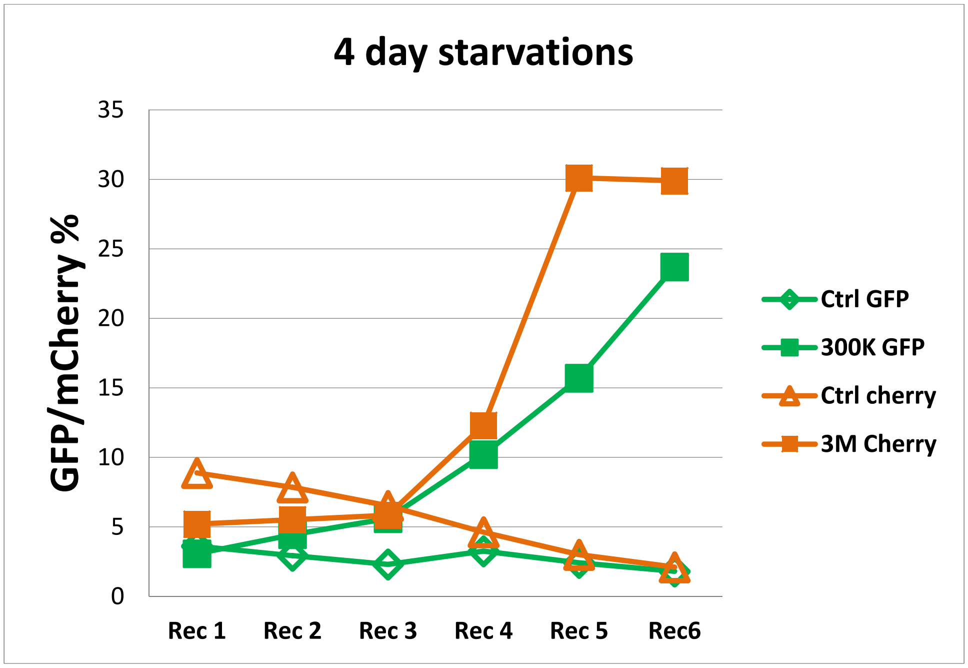

Figure S1. GFP and mCherry percentage after IL3 starvation/recovery cycles. FL5.12 cells were screened side-by-side with transduction of the first-generation (300K GFP) or second-generation (3M mCherry) library, along with the corresponding control shRNA. Cells were subject to IL3-withdrawal of three days (A) or four days (B). GFP or mCherry percentages after each recovery (Rec) are shown.
