# Supplementary material for: Introduction of Mismatches in a Random shRNA-Encoding Library Improves Potency for Phenotypic Selection
Source: PLoS One. 2014 Feb 3;9(2):e87390. doi: 10.1371/journal.pone.0087390 (PMC3911983; doi:10.1371/journal.pone.0087390)
Supplement: Figure S2 — shRNAs selected from the second-generation library better protect FL5.12 cells from IL3 withdrawal. FL5.12 cells were transduced with different shRNA clones obtained from the side-by-side screen. The cells were subject to an overnight IL3 withdrawal. Survival percentages (percentages of GFP+/To-Pro-3- cells) are shown, relative to the beginning of IL3 starvation. All six clones, five from the second-generation (3M) library and one from the first-generation (300K) library were significantly more protective than control (p<0.0001 for all). Clones 3M-3, -6, and -10 were also significantly more protective than clone 300K-5 (p<0.001 for all). (PDF) [file pone.0087390.s002.pdf]

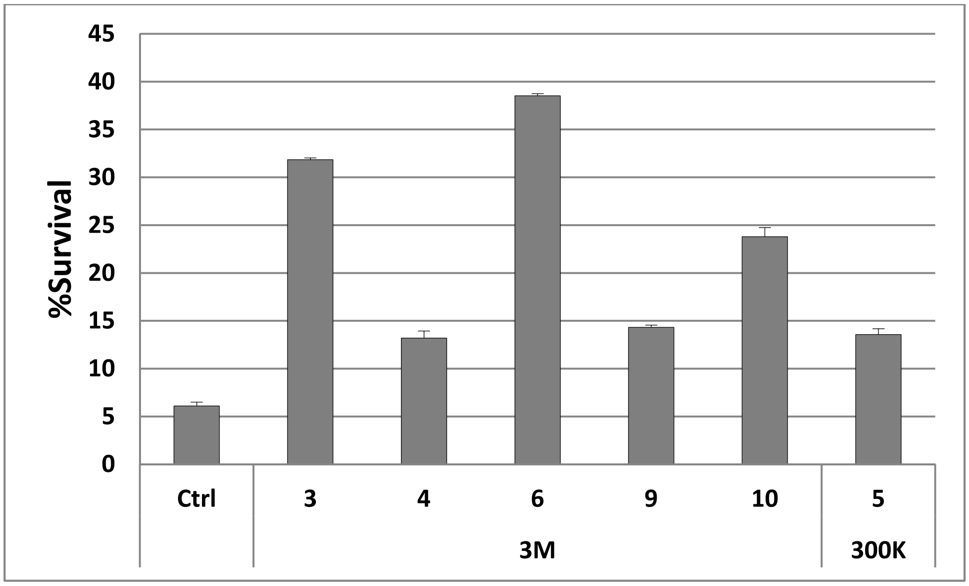

Figure S2. shRNAs selected from the second-generation library better protect FL5.12 cells from IL3 withdrawal. FL5.12 cells were transduced with different shRNA clones obtained from the side-by-side screen. The cells were subject to an overnight IL3 withdrawal. Survival percentages (percentages of GFP+/To-Pro-3- cells) are shown, relative to the beginning of IL3 starvation. All six clones, five from the second-generation (3M) library and one from the first-generation (300K) library were significantly more protective than control ($p<0.0001$ for all). Clones 3M-3, -6, and -10 were also significantly more protective than clone 300K-5 ($p<0.001$ for all).
